# Case Report: Primary myelofibrosis presenting with non-cirrhotic portal hypertension and initially misdiagnosed as cirrhosis

**DOI:** 10.3389/fmed.2026.1837166

**Published:** 2026-08-31

**Authors:** Liang Li, Yulu Chen, Junyi Chen, Guoxing Wu, Jingli Wen, Huichun Ji, Youde Yan

**Affiliations:** 1 Department of Infectious Diseases, The Suqian Clinical College of Xuzhou Medical University, Suqian, China; 2 Department of Infectious Diseases, The First Affiliated Hospital of Nanjing Medical University, Jiangsu Province Hospital, Nanjing, China

**Keywords:** anemia, cirrhosis, extramedullary hematopoiesis, portal hypertension, primary myelofibrosis

## Abstract

Primary myelofibrosis (PMF) is a rare cause of non-cirrhotic portal hypertension (NCPH). Because the clinical manifestations of NCPH often resemble those of cirrhosis-related portal hypertension, it is frequently misdiagnosed as cirrhosis in clinical practice. Herein, we report the case of a patient who presented with long-standing fatigue, progressive anemia, splenomegaly, and manifestations of portal hypertension, and was initially considered to have cirrhosis-related portal hypertension; a subsequent hematologic evaluation established the diagnosis of PMF. The patient then received standard ruxolitinib-based therapy, with improvement in spleen size and related symptoms. This case highlights that when the degree of liver injury is discordant with the severity of portal hypertension, particularly in the presence of hematologic abnormalities that cannot be fully explained by hypersplenism, potential underlying hematologic causes should be actively investigated.

## Introduction

Non-cirrhotic portal hypertension (NCPH) is a group of vascular liver diseases characterized predominantly by portal hypertension. Because it may present with clinical and imaging features similar to those of cirrhosis-related portal hypertension, such as splenomegaly and ascites, it is easily misdiagnosed as cirrhosis in clinical practice (1, 2). Primary myelofibrosis (PMF) is a myeloproliferative neoplasm and a rare hematologic cause of NCPH. When patients present with both features of portal hypertension and hematologic abnormalities, the diagnosis of an underlying hematologic disorder may still be delayed if the latter are not recognized as being beyond what can be explained by hypersplenism alone (3, 4).

A 68-year-old woman presented with long-standing fatigue and progressive anemia. Imaging studies demonstrated splenomegaly and portal hypertension, and she was initially considered to have cirrhosis-related portal hypertension; however, a subsequent hematologic evaluation revealed marked bone marrow fibrosis and a JAK2 V617F mutation, ultimately establishing the diagnosis of PMF-associated NCPH. This case suggests that when the severity of portal hypertension is disproportionate to the degree of hepatic parenchymal injury, particularly in the presence of hematologic abnormalities that cannot be adequately explained by hypersplenism alone, the possibility of NCPH should be considered, and potential underlying hematologic disorders should be actively investigated.

## Case presentation

On 7 March 2025, a 68-year-old woman presented with recurrent fatigue and poor appetite, accompanied by worsening abdominal distension and pain. She reported that fatigue and poor appetite had begun approximately 3 years earlier and that she had been diagnosed with cirrhosis at an outside hospital; however, the corresponding medical records and diagnostic basis were unavailable. She had received blood transfusions but no other specific treatment during that period. She had a history of gout and was treated with febuxostat. She denied chronic liver disease, viral hepatitis, harmful alcohol use, and other major medical conditions.

Seven months before admission, her fatigue and poor appetite worsened markedly, accompanied by right upper-quadrant abdominal distension and discomfort. An unenhanced abdominal CT performed at an outside hospital was interpreted as showing cirrhosis, splenomegaly, portal hypertension, and minimal ascites. No portal vein thrombosis was described, and the original images could not be retrieved for reassessment. In August 2024, she was hospitalized again at the outside hospital. Complete blood count showed an erythrocyte count of 2.58 × 10^12^/L, hemoglobin of 60 g/L, and platelet count of 143 × 10^9^/L. An abdominal ultrasonography showed hepatomegaly (oblique diameter, approximately 179 mm), marked splenomegaly (227 × 84 mm), cavernous transformation of the portal vein, and ascites. She was diagnosed with decompensated cirrhosis, portal hypertension, and severe anemia and received blood transfusion, diuresis, and supportive treatment.

On 17 February 2025, her symptoms worsened again. Complete blood count revealed an erythrocyte count of 1.54 × 10^12^/L, hemoglobin of 35 g/L, and platelet count of 58 × 10^9^/L. Despite symptomatic treatment, her symptoms did not reduce significantly, and her hemoglobin showed no obvious recovery.

On 7 March 2025, the patient was admitted to our hospital. A physical examination revealed pallor. The liver was palpable 1.5 cm below the costal margin, and the spleen was palpable approximately 4 cm below the umbilicus. There was no jaundice, peripheral edema, or shifting dullness. The white-cell count was 4.97 × 10^9^/L and remained within the laboratory reference range on repeated testing during the initial hospitalization. Neutrophils accounted for 81.0% (absolute count, 4.03 × 10^9^/L) and lymphocytes for 14.2% (absolute count, 0.71 × 10^9^/L). The erythrocyte count was 2.04 × 10^12^/L, hemoglobin was 53 g/L, the platelet count was 94 × 10^9^/L, and the mean corpuscular volume was 88.2 fL.

**Figure 1:**
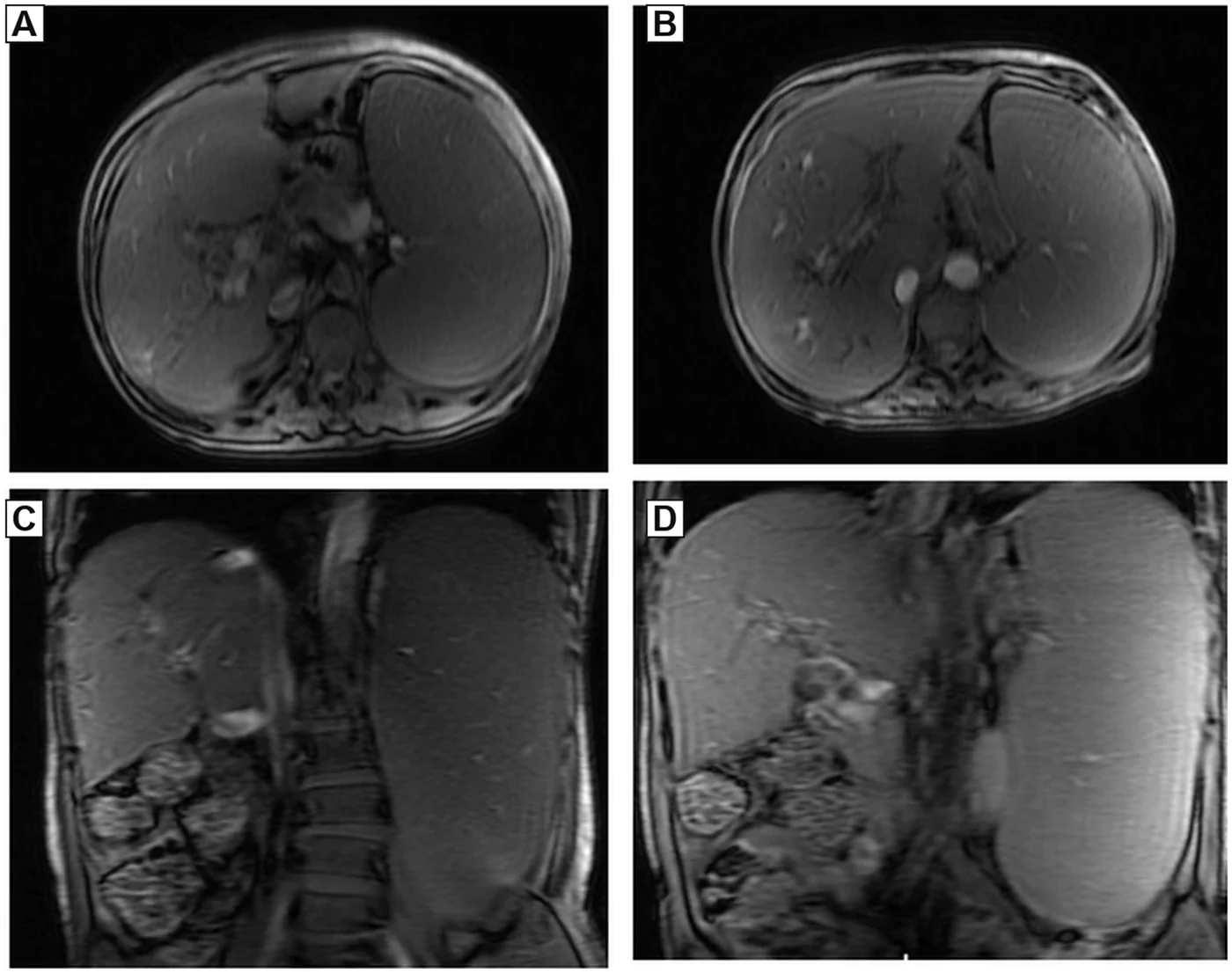
Upper abdominal MRI/MRCP showing portal-hypertensive changes. **(A,B)** Axial and **(C,D)** coronal images demonstrate marked splenomegaly, poor visualization of the main portal vein and its right and left branches, and cavernous transformation of the portal vein.

Coagulation testing showed a prothrombin time of 13.3 s, international normalized ratio of 1.13, activated partial thromboplastin time of 39.39 s, thrombin time of 15.20 s, fibrinogen of 3.70 g/L, and D-dimer of 0.230 mg/L; only the activated partial thromboplastin time was mildly prolonged. A biochemical analysis revealed a total bilirubin of 21.6 µmol/L, serum albumin of 36.9 g/L, alanine aminotransferase of 2.9 U/L, and aspartate aminotransferase of 8.8 U/L. Procalcitonin was 0.638 ng/mL, Golgi protein 73 was 320.00 ng/mL, and fecal occult blood testing showed a positive result. Serologic testing for hepatitis A and E virus IgM antibodies, ceruloplasmin, alpha-fetoprotein, carcinoembryonic antigen, autoimmune hepatitis-related antibodies, complement C3 and C4, antinuclear antibody profile, blood glucose, lipid profile, and routine urinalysis showed unremarkable results. A transient elastography demonstrated a controlled attenuation parameter of 186 dB/m and a liver stiffness measurement of 11.7 kPa. An abdominal magnetic resonance imaging/magnetic resonance cholangiopancreatography showed cirrhosis-like morphologic changes, marked splenomegaly, a small volume of ascites, and portal hypertension. The splenic parenchyma showed heterogeneous signal intensity, and the main portal vein and its right and left branches were poorly visualized, with cavernous transformation of the portal vein (Figure 1). No examination directly demonstrated an intraluminal portal vein thrombus. These findings were considered compatible with chronic portal venous obstruction, with prior portal vein thrombosis considered a potential cause (6). The patient-reported outside diagnosis of cirrhosis initially led us to consider cirrhosis-associated portal hypertension. However, liver biochemical abnormalities were limited, hepatic synthetic function was relatively preserved, only a small volume of ascites was present, and liver stiffness did not reach the diagnostic threshold for cirrhosis. In addition, the profound anemia, repeatedly normal white-cell count, and initially preserved platelet count were not fully explained by the usual hematologic pattern of cirrhosis-associated hypersplenism. This discrepancy prompted further hematologic evaluation.

**Figure 2:**
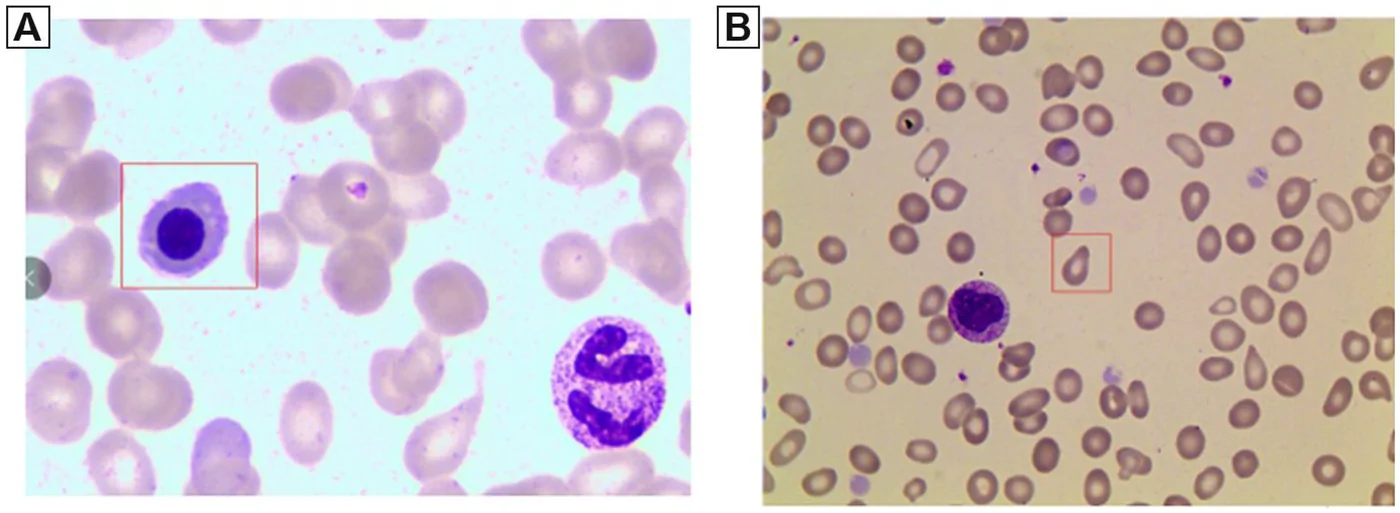
Peripheral-blood smear showing **(A)** a nucleated erythrocyte within the left box and **(B)** a teardrop-shaped erythrocyte within the right box.

**Figure 3:**
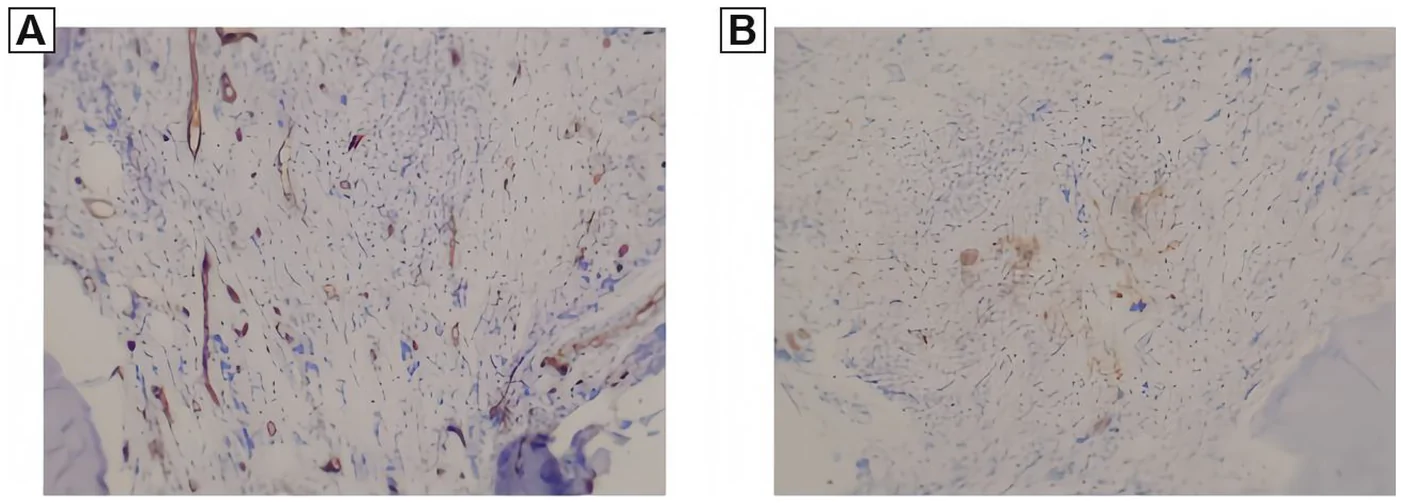
CD34 small vessels (+) with occasional round nucleated cells (+); CD117 (−); CD61 megakaryocytes scant (+) with occasional mononuclear cells; CD3 few (+); CD20 few (+); CD56 few (+); CD30 (−); CK (−); CD138 occasional (+). Reticulin staining: MF-3, consistent with primary myelofibrosis, fibrotic phase (reticulin MF-3, collagen fibrosis grade 3, osteosclerosis grade 0).

A peripheral blood smear examination demonstrated increased proportions of myelocytes and metamyelocytes, marked anisocytosis of mature erythrocytes, and scattered platelets. The left boxed cell was a nucleated erythrocyte, whereas the right boxed cell was a teardrop-shaped erythrocyte (Figure 2). A bone marrow aspiration revealed marked megakaryocytic proliferation with maturation abnormalities. A bone marrow biopsy demonstrated prominent fibrosis, with occasional identifiable megakaryocytes. Immunohistochemistry showed CD34-positive small vessels with occasional positive round nucleated cells, scant CD61-positive megakaryocytes, and occasional CD138-positive cells; reticulin staining was graded as MF-3, with collagen fibrosis grade 3 and osteosclerosis grade 0 (Figure 3). Myeloproliferative neoplasm genetic testing identified a JAK2 V617F mutation with a variant allele frequency of 53.4%. Based on the clinical manifestations, bone marrow morphology, pathology, and molecular findings, a final diagnosis of primary myelofibrosis was established.

**Figure 4:**
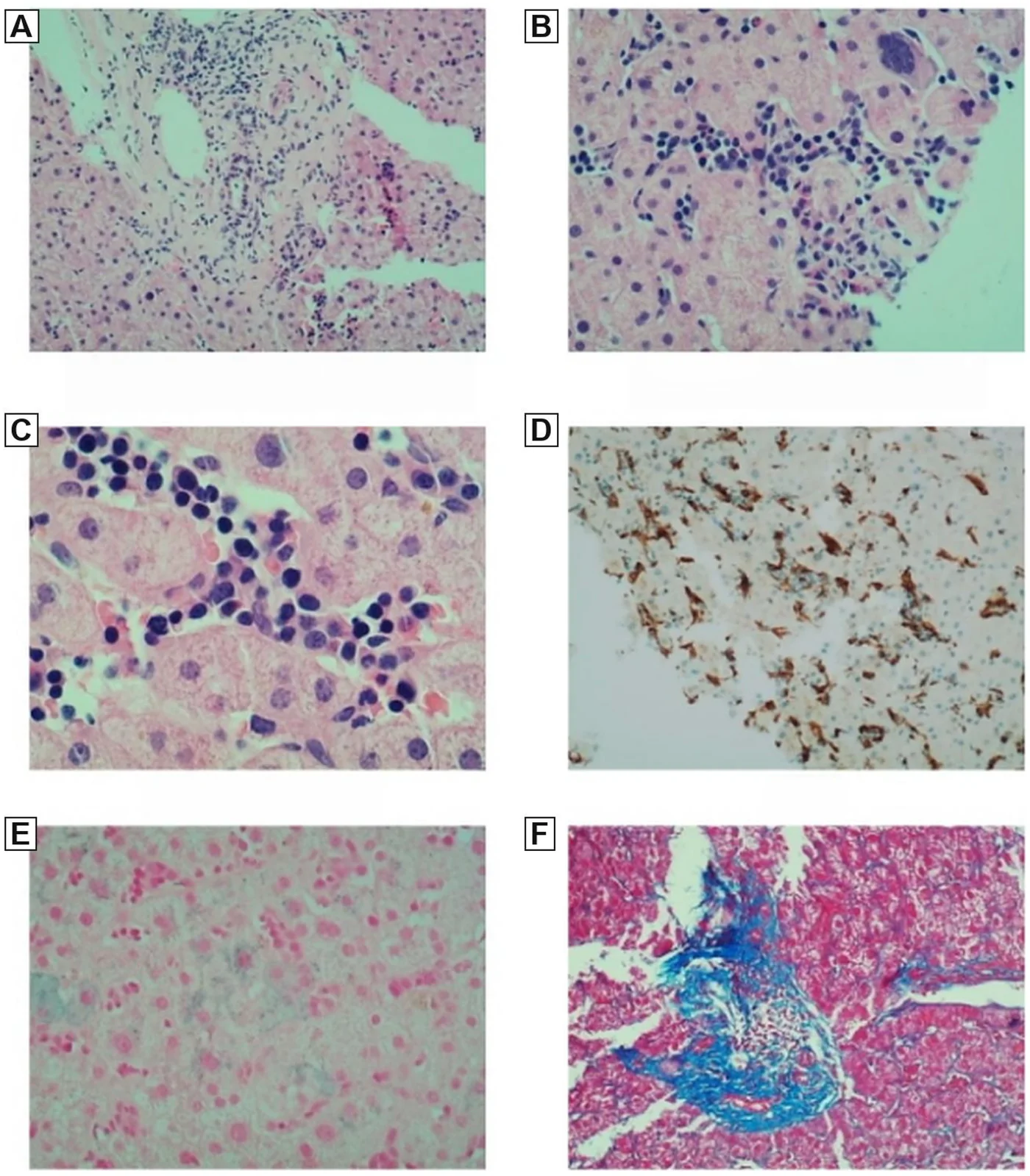
A liver biopsy demonstrating no cirrhotic architecture, but it revealed abnormal changes in portal vein branches and numerous nucleated cells aggregated within the hepatic sinusoids, indicating extramedullary hematopoiesis.

To further evaluate the hepatic pathologic changes, a percutaneous liver biopsy was performed (Figure 4). A histologic examination showed no cirrhotic architecture but revealed abnormal changes in portal vein branches and numerous nucleated cells aggregated within the hepatic sinusoids, indicating extramedullary hematopoiesis.

**Figure 5:**
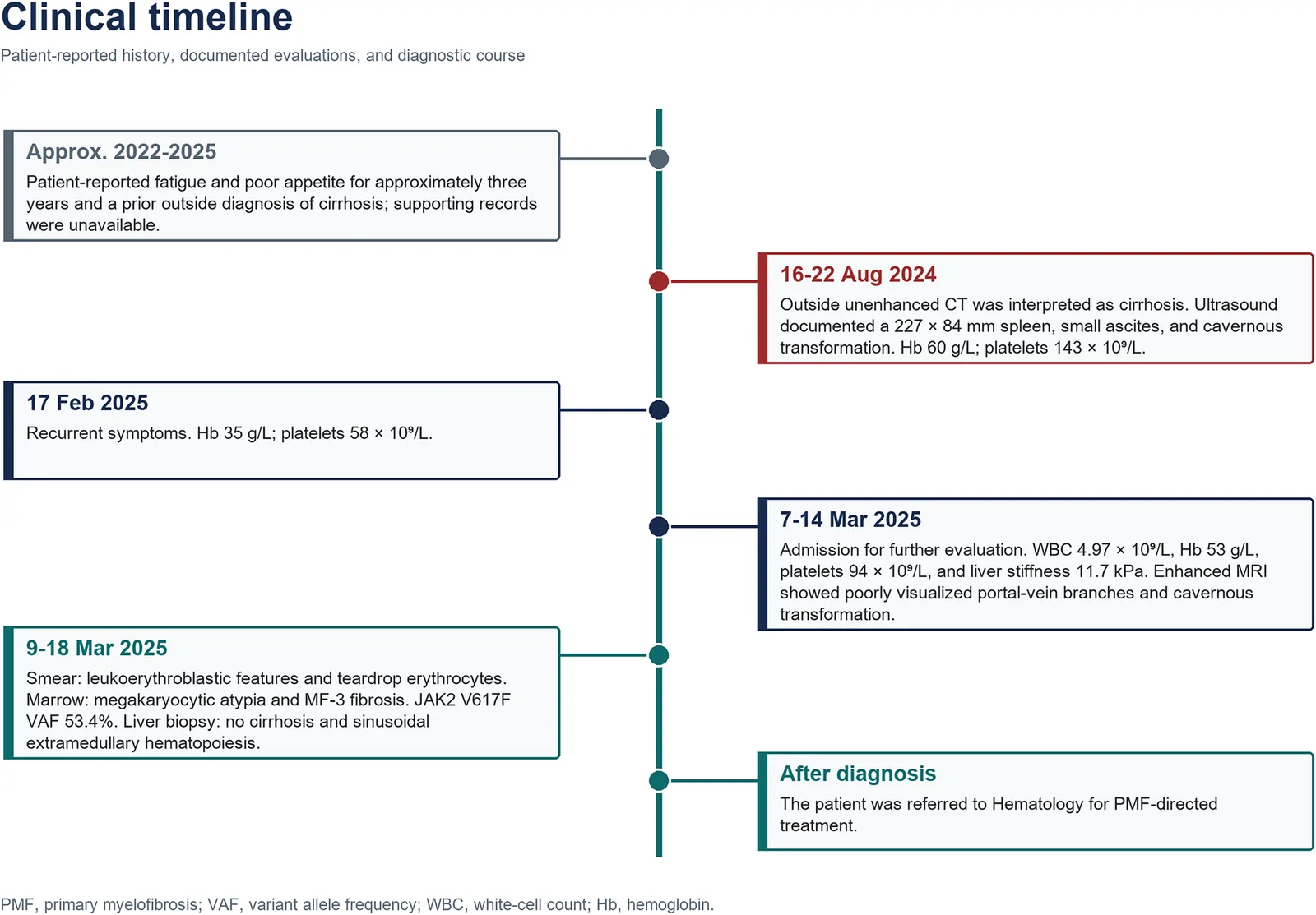
Patient-reported history, documented evaluations, and diagnostic course of the enrolled patient.

After the diagnosis was established, the patient was transferred to the Hematology Department and received targeted treatment centered on ruxolitinib, together with supportive therapy including testosterone undecanoate and recombinant human erythropoietin. During the 21–31 March admission, testosterone undecanoate 80 mg orally twice daily was administered. Ruxolitinib 10 mg orally twice daily and recombinant human erythropoietin 10,000 IU subcutaneously three times weekly were initiated on 28 March. By 31 March, the white-cell count, hemoglobin, and platelet count were 9.93 × 10^9^/L, 83 g/L, and 191 × 10^9^/L, respectively. Ruxolitinib was subsequently withheld after recurrent cytopenias. The patient was readmitted to the Hematology Department on 20 April with pancytopenia; the corresponding values were 2.78 × 10^9^/L, 46 g/L, and 26 × 10^9^/L. During this readmission, testosterone undecanoate and erythropoietin were continued, and repeated red-cell transfusions were administered. Thalidomide 50 mg nightly and prednisone 20 mg daily were started on 24 April, and ruxolitinib was restarted at 5 mg twice daily on 26 April. Ultrasonography on 22 April showed marked splenomegaly, with the spleen measuring 252 × 83 mm. By 28 April, the white-cell count, hemoglobin, and platelet count were 8.46 × 10^9^/L, 71 g/L, and 66 × 10^9^/L, respectively. A follow-up ultrasonography on 22 July showed persistent splenomegaly measuring 244 × 81 mm and a right-lobe oblique liver diameter of 164 mm. Because several disease-directed and supportive interventions were administered concurrently, neither the hematologic changes nor the small change in splenic dimensions could be attributed to ruxolitinib alone. Serial changes in blood counts during the diagnostic evaluation and early hematologic treatment are summarized in Table 1. The diagnostic course is summarized in Figure 5.

**Table 1:** Selected serial complete blood counts during diagnostic evaluation and early hematologic treatment.

Date	RBC (×10^12^/L)	WBC (×10^9^/L)	Hb (g/L)	Platelets (×10^9^/L)	MCV (fL)	Clinical context
22 August 2024	2.58	ND	60	143	ND	First documented outside-hospital CBC
17 February 2025	1.54	ND	35	58	ND	Outside-hospital readmission before red-cell transfusion
25 February 2025	2.25	5.75	61	61	ND	Anemia-clinic reassessment after outside-hospital treatment
7 March 2025	2.04	4.97	53	94	88.0	Diagnostic admission; before 1.5 U red-cell transfusion
10 March 2025	2.35	6.37	61	97	89.2	After transfusion during diagnostic admission
28 March 2025	2.32	6.14	65	109	86.8	Before same-day initiation of ruxolitinib and erythropoietin
31 March 2025	3.06	9.93	83	191	94.4	After 3 days of combined hematologic treatment
19 April 2025	ND	3.56	53	35	ND	Recurrent cytopenias after ruxolitinib had been withheld
20 April 2025	1.72	2.78	46	26	84.4	Readmission with pancytopenia; before 1.5 U red-cell transfusion
22 April 2025	1.72	3.26	47	34	87.7	After the 20 Apr transfusion; before a further 1.5 U transfusion
24 April 2025	1.95	4.53	54	48	87.7	Before 2.0 U transfusion and initiation of thalidomide/prednisone
26 April 2025	2.16	5.76	59	59	87.0	Before ruxolitinib restart and 2.0 U transfusion that day
28 April 2025	2.55	8.46	71	66	87.6	After repeated transfusions and combined treatment

ND, not documented; RBC, red blood cell count; WBC, white blood cell count; Hb, hemoglobin; MCV, mean corpuscular volume; CBC, complete blood count. Values were transcribed directly from the available records; MCV was not calculated when it was not reported.

## Discussion

NCPH is a vascular liver disorder characterized primarily by portal hypertension. Its diagnosis requires clinical or radiologic evidence of portal hypertension, such as splenomegaly, gastroesophageal varices, portosystemic collaterals, or ascites, while excluding cirrhosis, advanced fibrosis, and other chronic liver diseases that may cause portal hypertension (5). These findings support the diagnosis but are not individually specific. The portal and hepatic venous systems should also be assessed to identify prehepatic or posthepatic obstruction rather than assuming that portal vein thrombosis has been excluded (6). Because early clinical manifestations of NCPH are non-specific, patients are frequently misdiagnosed as having cirrhosis based on clinical presentation and imaging findings. However, in contrast to cirrhosis-related portal hypertension, hepatic synthetic and metabolic functions are relatively preserved in NCPH. Therefore, liver biopsy plays a critical role in distinguishing NCPH from cirrhosis, while low liver stiffness values on transient elastography (<12 kPa) may serve as an important non-invasive supportive indicator (7).

Although the precise etiology of NCPH remains unclear, it has been associated with prothrombotic conditions, immune-mediated diseases, chronic infections, exposure to certain drugs or toxins, and genetic susceptibility. Histopathologic features most commonly include portal vein sclerosis, narrowing or obliteration of portal vein branches, sinusoidal dilatation, nodular regenerative hyperplasia, paraportal shunt vessels, and perisinusoidal fibrosis, supporting a predominantly vascular pathogenesis rather than primary hepatocellular injury (8, 9).

In the present case, cavernous transformation of the portal vein and a small volume of ascites were imaging findings consistent with portal hypertension. Massive splenomegaly was supportive but not specific because PMF-related splenic extramedullary hematopoiesis may independently cause marked splenic enlargement. Because the most common cause of portal hypertension in clinical practice is increased sinusoidal resistance secondary to cirrhosis, she was initially diagnosed with cirrhosis-associated portal hypertension. However, further evaluation showed that liver biochemical parameters were largely unremarkable, with only mild hyperbilirubinemia, and hepatic synthetic function was relatively preserved. Imaging studies revealed only a small amount of ascites, and liver stiffness did not reach the diagnostic threshold for cirrhosis. In compensated cirrhosis, thrombocytopenia is generally the earliest and most common hematologic abnormality, followed by leukopenia and anemia (10). By contrast, this patient had profound progressive anemia with a repeatedly normal white-cell count and initially preserved platelets. The leukoerythroblastic blood picture, teardrop erythrocytes, megakaryocytic atypia, and MF-3 marrow fibrosis could not be explained by hypersplenism alone and prompted the hematologic evaluation that established PMF.

PMF is a chronic myeloproliferative neoplasm characterized by marked proliferation of megakaryocytes and granulocytes, reactive fibrous tissue deposition in the bone marrow, and extramedullary hematopoiesis (11). PMF represents a rare underlying cause of NCPH, and its pathogenic mechanisms remain debated. Current evidence suggests several contributing factors: (1) extramedullary hematopoiesis within the spleen and massive splenomegaly may lead to a hyperdynamic circulatory state, significantly increasing blood flow into the portal venous system and promoting portal hypertension; (2) hepatic extramedullary hematopoiesis or sinusoidal architectural alterations may increase intrahepatic vascular resistance, resulting in elevated portal pressure in the absence of cirrhotic structural changes; and (3) the prothrombotic state associated with PMF increases the risk of splanchnic venous thrombosis, and portal venous system thrombosis itself is a recognized cause of NCPH (12). Available data, although limited, suggest that portal hypertension develops in a clinically meaningful minority of patients with myeloproliferative neoplasms. In a mixed cohort of 29 patients, portal hypertension was identified in 4 (13.8%), including 2 (6.9%) with esophageal and/or fundal varices (13). Similarly, a retrospective series of 123 patients with myelofibrosis with myeloid metaplasia identified portal hypertension in 13 (10.6%) (14). Although based on small and heterogeneous cohorts, these findings highlight the clinical relevance of recognizing portal-hypertensive complications, including gastroesophageal varices, in patients with myeloproliferative neoplasms (MPNs) (15).

Accordingly, when clinical manifestations of portal hypertension are disproportionate to the degree of hepatic parenchymal injury, particularly in the presence of unexplained or disproportionate hematologic abnormalities, clinicians should consider vascular, thrombotic, or hematologic causes of NCPH.

## Data Availability

The original contributions presented in the study are included in the article/Supplementary Material, and further inquiries can be directed to the corresponding author.
